# Genetic Diversity on Farm in Japanese Paper Mulberry

**DOI:** 10.1002/ece3.70828

**Published:** 2025-01-09

**Authors:** Dinh Thi Lam, Katsunori Tanaka, Akihiko Takashima, Ayako Shibutani, Ryuji Ishikawa

**Affiliations:** ^1^ Faculty of Agriculture and Life Science, Hirosaki University Hirosaki Aomori Japan; ^2^ Historiographical Institute The University of Tokyo Bunkyo‐ku Tokyo Japan

**Keywords:** *Broussonetia*, chloroplast genome, MIG‐seq, paper mulberry

## Abstract

Paper mulberry is a fiber resource for paper making. Washi, a traditional paper in Japan, has been produced from *Broussonetia* × *kazinoki*, a hybrid between *B. monoica* and *B. papyrifera* . Elite strains have been vegetatively propagated and distributed within Japan. In this study, these three strains' complete chloroplast (cp) genomes were reconstructed as pseudomolecules consisting of 160,861, 160,868, and 160,878 bp, respectively. They were compared with other database strains to detect insertion/deletion (INDEL) polymorphisms. In total, 25 INDELs were identified in these cp genomes. ISSR‐based MIG‐seq polymorphisms were applied to determine whether varieties or regional groups can be discriminated. Although various types of cultivars exhibiting different appearances are hard to discriminate, principal component analysis (PCA) distinguished two major groups. Maternal lineages reflecting the diverse origins of Japanese paper mulberry were determined using hypervariable maternal markers. An NGS‐oriented nuclear marker system revealed the heterogeneous nature of domesticated paper mulberry, reflecting the complex origins of Japanese *B.* × *kazinoki*.

## Graphical abstract

Genetic diversity in Japanese paper mulberry was confirmed with MIG‐seq followed by PCA. Including escaped type domesticated plants, there are a few groups that were identified with hyper‐variable maternal lineages. The genetic diversity are observed on farms.

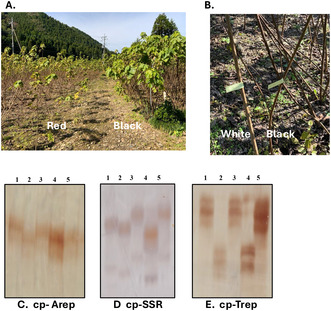

## 1 Introduction

The moraceous tree species paper mulberry ( *Broussonetia papyrifera* ) is a well‐known valuable tree species used as a fiber resource in the paper industry. The *Broussonetia* comprised three species, *B. kaempferi* Siebold, *B. monoica* Hance, and *B. papyrifera* (L.) L'Hér. ex Vent. which is found across Southeast and East Asia and the Pacific islands (Chang et al. 2015; Chung et al. 2017).

Paper‐making techniques in Japan were introduced from the Asian continent through the Korean peninsula, but details of the period when the tree was introduced into Japan are unclear. The period was noted in a Japanese historical book, the Nihon Shoki (The Chronicles of Japan), dated AD610 from China through the Korean peninsula. Japanese traditional paper, Washi is made from three plant materials, including *Wilstroemia sikokiana*, known as “Gampi” in Japanese, have subsequently been used to make white paper, *Edgeworthia chrysantha* , called “Mitsumata” in Japanese, was introduced from East Asia, and “Kozo” which is the main source of fiber for making Washi.

Kozo is derived from several species including hybrids. The four species of *Broussonetia* in Japan are *B. kaempferi* (Tsuru‐kozo), *B*. *monoica* (Himekozo), *B. papyrifera* (Kajinoki), and *B*. × *kazinoki*, known as Kozo, which is a hybrid between *B. monoica* and *B. papyrifera* , as determined in several phylogenetic studies (Ohba and Akiyama 2014; Chung et al. 2017). The hybridization happened unidirectionally with *B. monoica* as the maternal donor (Wong 2019; Kuo et al. 2022). Nuclear ribosomal DNA polymorphism and maternal sequence analysis demonstrated that hybridization occurred for Kozo in Japan and multiple times for Daknamu in Korea (Kuo et al. 2022). Despite this genetic background, morphological appearances suggested that there is wide variation among Kozo, which was recorded in the Encyclopedia of Agriculture “Nogyo‐zensho” (Miyazaki 1697; Okura 1946). Japanese traditional paper has been made with Kozo and sometimes *B. kaempferi* (Tsuru‐kozo), *B. monoica* (Himekozo), and *B. papyrifera* (Kajinoki). The four species have been used not only as material for paper production but also for their leaves as a tea.

There are several landraces of Kozo, such as Aoso, Akaso, Kaname, Nasu, Taori, and others known by different local names. Nasu produced originally in Ibaraki prefecture, located in the middle of Japan, is regarded as a high‐quality source for Washi paper making and has been grafted for distribution to other areas to keep its quality as an elite line for paper manufacturing. As the import of paper from Southeast Asian countries has increased gradually, domestic products cannot compete because of their higher cost and local production is decreasing. Farmer closed their farms causing paper mulberry production in Japan to shrink. Nevertheless, the farms consequently turned to be redundant; there are many redundant Kozo plants from such farms found nowadays in nearby secondary forests. To understand whole variation of kozo, not only conserved landraces but also escaped kozo are required to survey their genetic diversity.

Japanese traditional washi paper is generally produced not only with kozo but also with starches from rice, a sticky ingredient from Tororo‐aoi ( *Abelmoschus Manihot* ). Microscopic analysis detected starch granules with fibers from aged washi papers (Shibutani et al. 2022). These remains allowed researchers to recover aged DNA by amplification (Tanaka et al. 2015; Muto et al. 2020). In these trials, the aged DNA recovered from ancient rice grains were applied to estimate their origins. Remains of kozo and other plant species are also available to estimate their origins if sufficient molecular polymorphisms as tools are understood. Tracing origins of plants as fiber resource would work to recover which types of landraces had been applied to produce the paper. Washi paper had been used to keep important records in the past. The Shogun, Emperor, and even local lords used washi to record historical events such as tsunamis or earthquakes and to register tax payments etc. Because of deterioration of such documents, they are under‐conservated as historical evidence, however, are easily degraded. To protect these valuable historical documents, the use of identical materials is better for restoration. Preliminary studies have examined the fibers and tried to identify fine components in old papers under microscopy with high resolution as a non‐destructive survey (Shibutani et al. 2021; Kuo et al. 2022). Through the process, various starch granules possibly containing plastid DNA were observed. As there are various efforts to amplify aged DNA, such tools are required to identify the types of paper mulberry for restoration of historical documents. However, there are no data on whether paper mulberry has sufficient diversity to be able to identify the original locations where particular paper resources were produced and whether it is technically possible to determine any such differences.

In order to identify the original compositions of historical paper materials, which enables the reconstruction of the origin of paper materials that may lead to the interpretation of the historical and environmental background of documents, in this study, we conducted: (1) examination of the current status of original historical material Kozo in Japan, (2) analysis of the sequence data of chloroplast DNA in material plants to develop DNA markers that can be applied in tracing maternal lineage among the landraces, and (3) evaluation of nuclear genetic diversity by MIG‐seq and PCA to determine the paper materials originating from various regions and within single fields of cultivated paper mulberry plants in Japan.

## 2 Materials

### 2.1 Plant Materials

Samples of *B*. × *kazinoki* were collected in Japan, which were accompanied by GPS code and prefecture names in Japan (Table S1). In total, 52 samples were applied for chloroplast polymorphism screening. Forty‐eight samples carrying various plastid types were applied to MIG‐seq analysis. Multiple samples were collected from single fields. Eight samples (four with black bark, one with white bark, and three with red bark) from Farm TK, and 17 samples from Farm T were randomly collected from a farm in Ibaraki prefecture. All samples were kindly donated by owners possessing samples and some were collected from publicly available sites where is not any private lands or conserved areas. Most of them were along public road.

### 2.2 DNA Analysis

Registered paper mulberry chloroplast genomes, KX828844.1 (160,239 bp; *B. papyrifera* Korea) and NC_037021 (160,903 bp; *B. kazinoki crossed B. papyrifera*) were aligned to create chloroplast INDEL markers. Three primer pairs were further applied to evaluate maternal lineages among Japanese paper mulberry samples. DNA was extracted using a DNeasy plant kit (QIAGEN Co., Japan). P5000 Taq polymerase (Agilent, Japan) was applied with a company‐supplied buffer system. INDEL markers, then, were amplified with PCR cycles by preheating at 94°C for 3 min., 30 rounds of 94°C 5 s., 50°C for 30 s., and 72°C for 30 s. The amplified products were electrophoresed in 6% sequencing gels and stained with silver nitrate to detect polymorphisms. All primers are listed in Table S2.

### 2.3 Assembly of Complete Chloroplast Genomes

We obtained 100‐bp pair‐end reads of landraces Aoso, Kaname, and Taori using an Illumina HiSeq 2500 platform (Illumina, Japan). The resulting 4516; 4571 and 4620 Mb of sequences for Aoso, Kaname, and Taori comprised 44,713,896, 45,255,920, and 45,746,220 reads, respectively. CLC genomic workbench ver. 21 was used to make *de novo* assemblies. Contigs with high coverages compared with other contigs were used to assemble pseudo chloroplast genomes for the three landraces. Pseudomolecules with annotations in GenBank format were applied using Organellar Genome DRAW into graphical maps (Greiner, Lehwark, and Bock 2019).

### 2.4 MIG‐Seq

DNA was extracted using a DNeasy plant kit (QIAGEN Co., Japan). In total, 10 ng of genomic DNA was applied for multiplex PCR, In the first PCR step, 3.5 μL 2× Multiplex PCR buffer, 0.7 μL primer mix, 1 μL genomic DNA (10 ng), 0.035 multiplex PCR enzyme, and 1.765 μL H_2_O. PCR running conditions were: 94°C 1 min, 27 cycles of 94°C 1 min, 38°C 30 s, and 72°C 1 min, and 72°C for 10 min as a final reaction. The second PCR was conducted with 4 μL 5× PS buffer, 1.6 μL 2.5 mM dNTPs, 2 μL 10 μM forward primer, 2 μL 10 μM reverse primer, 2 μL diluted first PCR product including 5–10 ng amplified DNA, 0.2 μL Prime Star polymerase, 8.2 μL H_2_O. Experimental conditions were as described by Suyama and Matsuki (2015).

### 2.5 Data Analysis

MIG‐seq products were sequenced by DNBSEQ G400 to obtain 2 × 100‐bp reads. Adapter sequences were removed using Cutadapt (ver. 1.9.1). Low‐quality reads were removed using Sickle (ver. 1.33), when the quality score was less than 20 and removed reads less than 50 nt in length. SNP in reads were extracted using denovo_map.pl. in Stacks (ver.2.41).

## 3 Results

### 3.1 Maternal Diversity

**FIGURE 1:**
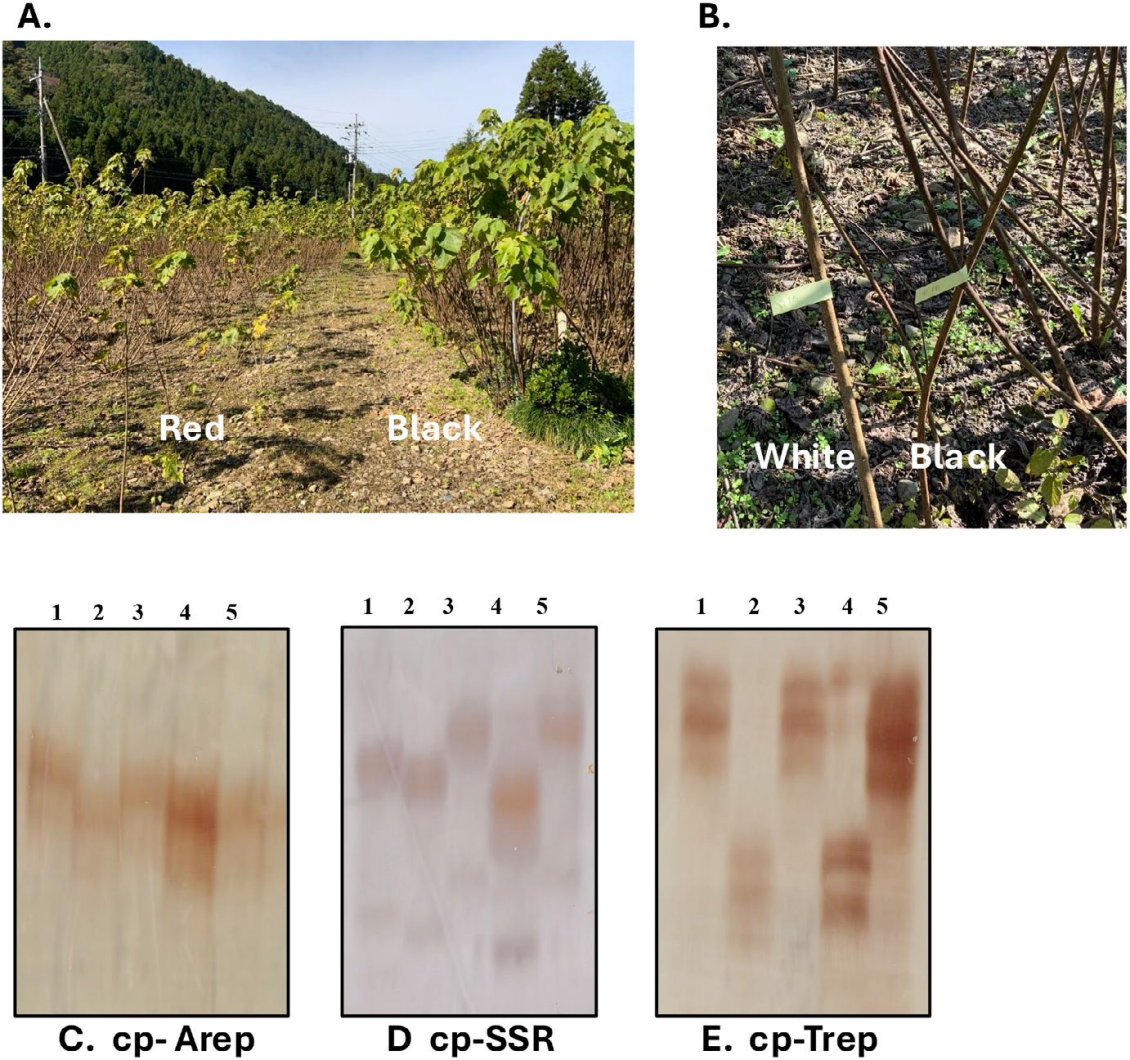
Cultivated kazinoki at Taishi, Ibaraki prefecture. (A) Left‐side group Aka‐kozo (red bark) and right‐side group Kuro‐kozo (black bark). (B) White bark kozo emerged from black bark kozo. (C) Variation of maternal type distinguished with cpINDEL plastid type from lanes 1 to 5; Aoso, Akaso, Kaname, Nasu, and Taori as elite strains. cp‐Arep. (D) cp‐SSR. E. Trep. From lanes 1 to 5; Aoso, Akaso, Kaname, Nasu, and Taori as elite strains.

Both complete chloroplast genomes, KX828844.1 (160,239 bp, *B. papyrifera* ) and NC_037021 (160,903 bp, *B. kazinoki × B. papyrifera*) were aligned together to identify INDEL polymorphisms to detect maternal lineages. Three of several primer pairs designed amplified clear polymorphic patterns in 6% denatured sequencing gels (Figure 1, Table 1, Table S2). The polymorphisms were used to evaluate chloroplast genome diversity. Combinations of the polymorphic patterns allowed 15 chloroplast genome types to be distinguished. Four different maternal lineages were detected in multiple samples collected in a single farm, Ibaraki prefecture. Predominated elite landraces Aoso, Kaname, Nasu, and Taori showed that they belonged to different chloroplast genome types. There was no clear trend based on the geographical origins.

**TABLE 1:** Maternal lineages evaluated from cpINDELs.

Origin	No. of samples	Maternal type															Remarks
		M1	M2	M3	M4	M5	M6	M7	M8	M9	M10	M11	M12	M13	M14	M15	
Kyoto	1	1															Wild‐Escape
Kochi	5				2					1					1	1	Cultivar
Toyama	14					3			11								Cultivar
Kumamoto	2												2				Wild‐Escape
Hyogo	16		5	1								6	1			3	Wild‐Escape
Saga pref	6													6			Cultivar
Ibaraki	7						3	2		1						1	Cultivar
Ibaraki	1															1	Wild‐escape
Total	52	1	5	1	2	3	3	2	11	2	0	6	3	6	1	6	

Chloroplast genomes are maternally inherited in *Broussonetia* (Zhang et al. 2003). Multiple maternal haplotypes detected suggested that they resulted from asymmetrical hybridization or hypervariable markers (Tables S1 and S3). It was not only due to hypervariable markers, but also different origins of maternal lineages that might increase diversity among chloroplast types.

### 3.2 Complete Chloroplast Genomes

Three traditional paper mulberry landraces belonging to different maternal lineages were applied to get whole‐genome sequence 100‐bp pair‐end reads. A *de novo* assembly method was applied. The assembly of Aoso resulted in 2,540 contigs in total ranging from 4,999 to 85,366 bp in size with average an 8,873 bp. Average coverages ranged from 6.6 to 21,754. Longer sequence contigs with higher coverages of more than 2000 showed high similarity with the chloroplast genome sequence of rice ( *Oryza sativa* , XP15091.1), suggesting that the contigs were composed from cp genome sharing higher similarity among plant species. However, there were gaps between the contigs. Thus, whole reads of three landraces were applied for *de novo* assembly to create pseudomolecules divided into three contigs. The largest one was 95,303 bp in size, which corresponded to the large single copy (LSC) region. It showed a coverage of 7,249. The intermediate contig was 27,621 bp in size corresponding to the inverted repeat, with a coverage of 11,791, corresponding to nearly 1.5× compared with the LSC region. The shortest contig was 20,233 bp in size with a coverage of 8,523, corresponding to the short single‐copy (SSC) region. Small gaps, SNPs, and INDELs were filled with complementary data from reads. These pseudomolecules were applied as reference sequences to obtain pseudomolecules corresponding to each landrace. Re‐sequencing to the pseudomolecules resulted in landrace‐specific chloroplast genomes. Finally, three complete chloroplast genomes were obtained for Aoso, Kaname, and Taori, which were 160,868, 160,856, and 160,878 bp, respectively. Inverted repeats of the three genomes were 25,801, 25,805, and 25,802 bp, respectively (Table 2).

**TABLE 2:** Nine chloroplast genome sequences.

Accession	Size	LSC	INV	SSC	INV	Specimen	Reference Genbank accessions
Aoso	160,868	1–89,184	89,185–114,985	114,986–135,076	135,077–160,868	*Broussonetia kazinoki*	This paper
Kaname	160,861	1–89,179	89,191–114,995	114,996–135,056	135,057–160,856	*Broussonetia kazinoki*	This paper
Taori	160,878	1–89,182	89,184–114,986	114,987–135,075	135,076–160,878	*Broussonetia kazinoki*	This paper
NC_037021	160,903	—	—	—	—	*Broussonetia kazinoki*	GenBank record
NC_059692.1	160,861	—	—	—	—	*Broussonetia kazinoki*	GenBank record
MH223642.1	160,841	—	—	—	—	*Broussonetia kazinoki*	GenBank record
MW465960.1	160,841	—	—	—	—	*Broussonetia kazinoki*	GenBank record
MF496038	160,903	—	—	—	—	*Broussonetia kazinoki*	GenBank record
KX828844.1	160,239	—	—	—	—	*Broussonetia papyrifera*	GenBank record

**FIGURE 2:**
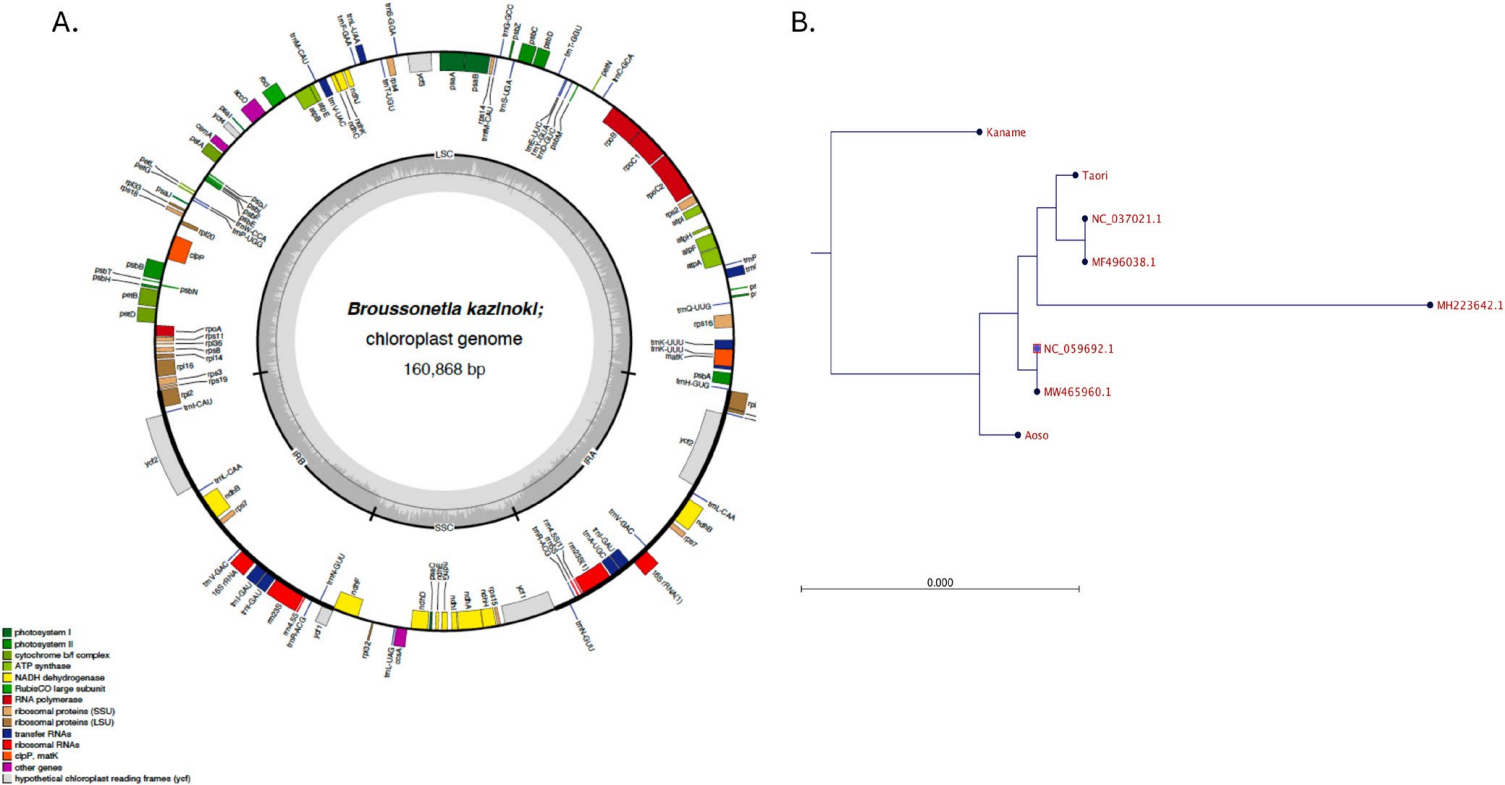
Map of the complete whole chloroplast genome of Aoso. Presumed genes are noted in a clockwise direction. (A) Annotations referenced from other cp genomes. Genes with reverse transcriptional directions noted in an anticlockwise direction. (B) Phylogenetic tree constructed using the neighbor‐joining method and measured nucleotide distances using the Jukes–Cantor method (replicates were performed 1,000 times for bootstrap analysis).

A phylogenetic tree using whole chloroplast genomes with *B. papyrifera* indicated that Kozo genomes were positioned close together. In the next step, when *B. papyrifera* was excluded, Kaname was positioned far from the others (Figure 2, Figure S1.). One Kozo sample, MH223642.1, was distant from the others.

INDELs were detected among these three landraces (Table 3). There were several cases of INDEL polymorphisms including A/T repeats, SSR with TG or AATT as motifs, or non‐unique motifs. Corresponding sites to the three previous markers cp‐Arep, cp‐Trep, were cp‐SSR were identified in Aoso, Kaname, and Taori genomes. However, two of these markers, cp‐Arep and cp‐Trep, did not correspond to the result obtained by PCR amplification and gel electrophoresis (Table S3). It might result in applied individuals and polymorphism among single landraces. However, these presumed polymorphisms may work for additional markers for evaluation of genetic diversity, for traceability, or for screening elite strains.

**TABLE 3:** Chloroplast sequence variations composed from nine deletions and 14 insertions against Aoso genome among three landraces cultivated in Japan.

Region (Aoso)	INDEL type	Reference	Kaname	Taori	Remarks
1901..1911		—	—	—	^a^
5908^5909	Insertion	—	TAGATTA	—	
7890..7921		—	—	—	^a^
9351^9352	Insertion	—	—	A	A repeat
9377^9378	Insertion	—	—	AT	AT repeat (SSR marker)^a^
13436..13440	Deletion	TTTTT	—	—	
13685^13686	Insertion	—	T	—	T repeat
58976^58977	Insertion	—	T	—	T repeat
76561^76562	Insertion	—	T	TT	T repeat
102537^102538	Insertion	—	T	T	T repeat
105837^105838	Insertion	—	—	G	
105838..105839	Deletion	TG	—	—	TG repeat
117317^117318	Insertion	—	A	—	
117324^117325	Insertion	—	—	A	
117946..117951	Deletion	AATTAA	—	—	AATTAA repeat
118107^118108	Insertion	—	AAAAAAAA	—	A repeat
118363^118364	Insertion	—	—	TT	T repeat
118774^118776	Insertion	—	—	TGTTTCTAATTGGA	
119019..119026	Deletion	TTAGAATT	TTAGAATT	—	
119102	Deletion	A	—	A	
119104..119114	Deletion	GTATTAATATA	—	GTATTAATATA	
119116^119122	Deletion	ATATATA	—	ATATATA	AT repeats
127323	Deletion	A	A	—	
144233..144234	Deletion	CA	—		
147536^147537	Insertion	—	—	C	C repeat

^a^ Two INDELs detected between two other genomes deposited in NCBI are not detected as polymorphisms among these three genomes. An SSR marker represented polymorphism.

### 3.3 MIG‐Seq and PCA

**FIGURE 3:**
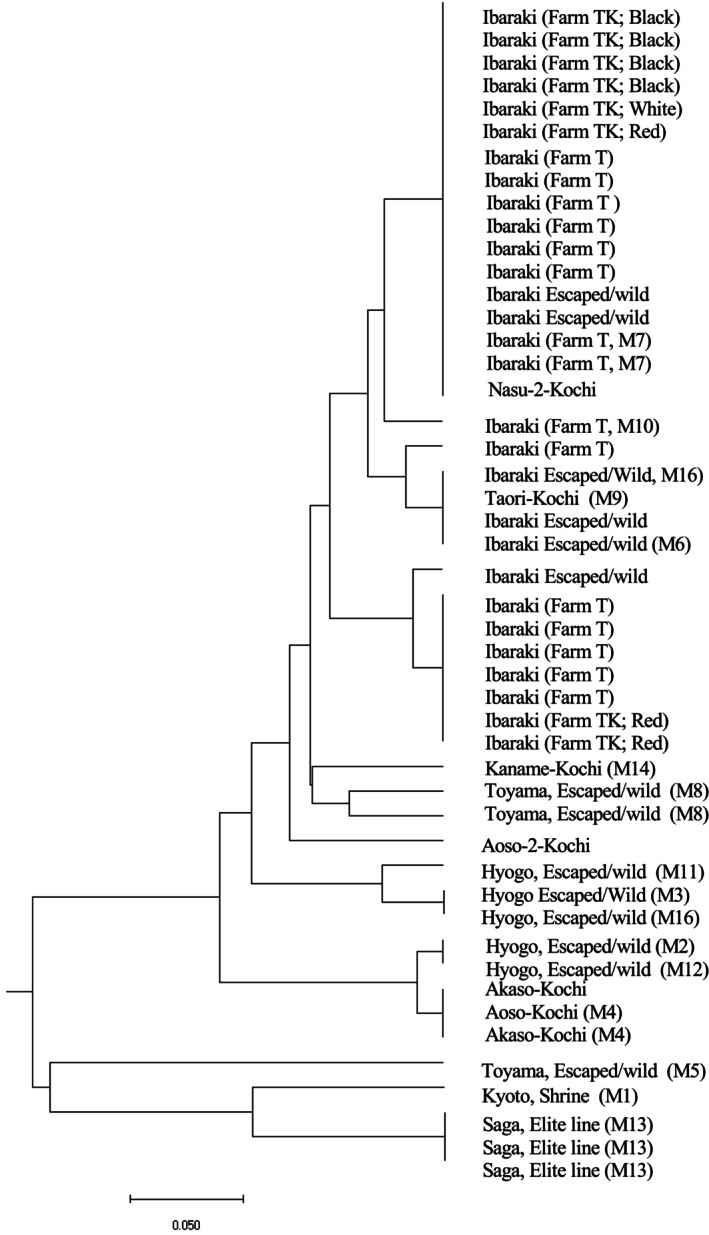
Phylogenetic tree constructed using UPGMA with 42 MIG‐seq polymorphisms. M in parenthesis refers to maternal lineages obtained using cpINDELs.

**FIGURE 4:**
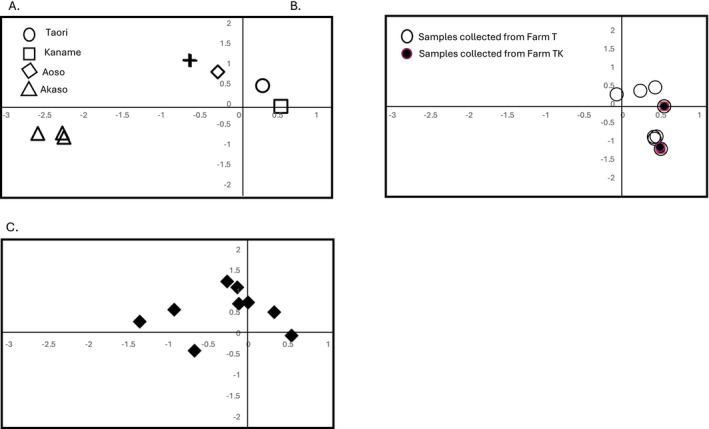
PCA with 42 polymorphic MIG‐seq genotypes. (A) Elite strains: circle; Taori, square; Kaname, diamond; Aoso, plus; Akaso. triangle; varieties in Saga prefecture. (B) Cultivars from farms: Solid circles; Farm TK, open circles; Farm T. (C) Escaped/wild plants.

Individuals with different maternal lineages were preferentially chosen to determine their nuclear genetic diversity. Additional strains from cultivated strains were applied to MIG‐seq analysis. This produced 13,777 amplicons among 48 accessions. However, a disadvantage of MIG‐seq can be a lack of data among huge numbers of polymorphic amplicons. The reason for null amplicons includes not being able to decide whether this is due to mis‐amplification or absence. Because of this, datasets including null types were strictly excluded across the huge datasets. As a result, 42 SNP loci were applied for further analysis to construct a phylogenetic tree and for PCA (Figures 3 and 4).

Individuals with the same maternal lineages such as M4 and M13 tended to locate with each other. Other lines with maternal datasets were scattered in the tree. Two individuals known as elite lines, Aoso, belonged to different clades. One of them was classified into the same clade with Akaso. Five individuals collected from a single farm, Farm TK, with various bark colors belonged to a single clade with eight cultivars from Farm T. However, several individuals from both farms were scattered in different clades. For example, individuals with red‐colored bark from Farm TK belonged to different clades. When we applied all the datasets, a similar trend was observed, but data plots were relatively scattered because of null genotypes (data not shown). Cultivars with different color barks, red, black, and white, were recognized from Farm TK.

In the PCA, axes 1 (25.6% contribution to total variation) and 2 (17.8%) were plotted as shown in Figure 3. Other axes were not contributed well. It was reflected that they had not diverged from each other, although many loci with absences of data were excluded. It resulted in overlapped plots with multiple samples. Elite lines formed two groups. One of them, collected from Saga prefecture, formed distinct plots (Figure 3A). Individuals from Farm TK and Farm T were included in one of the two groups distinct from materials from Saga prefecture (Figure 3B). Most escaped/wild individuals except for three were plotted close to Aoso, Akaso, Kaname, and Taori elite lines (Figure 3C). The three individuals were located between this group and another group collected in Saga prefecture.

## 4 Discussion

Genetic variation in plant species results from their origins, propagation, isolation, and distribution. Compared with wild species, genetic variation of domesticated plants is mainly affected by artificial management through the domestication process. In order to evaluate Japanese paper mulberry, we tried to understand genetic variation with cp genome data and novel nuclear marker system, MIG‐Seq. Previously, huge genetic variation in Southeast Asia has been reported, and then, a particular subgroup had been brought to remote oceanic islands for various usages (Chang et al. 2015). Paper mulberry is mainly utilized for paper making in Japan, followed by Mitsumata and Ganpi, which contain abundant fibers in the plant body. Valuable lineages as landraces are propagated artificially by vegetative propagation to produce superior paper. Kozo or Kajinoki in Japan are resources for paper making, which are hybrid between *B. papyrifera* and wild species *B. monoica* in Korea and Japan (Kuo et al. 2022). Hypervariable *ndhF‐rpl32* haplotypes suggested that there are several maternal lineages, which indicated that several crossings had occurred. In this research, we screened predominant paper mulberry as elite landraces, and escaped/wild individuals from secondary forests. As with the decline of the industrial sector, farming has decreased, and escaped paper mulberry lines are easily detected in secondary forests.

The chloroplast genome sequences provided markers to evaluate maternal lineages. Huge variations were observed. Various plastid types suggested that multiple hybridizations with natural populations of *B. monoica* may have contributed. No trend in distribution was observed due to the past distribution of elite strains (Okura 1946). In 1844, at least 16 landraces were recognized, and vegetative organs (tillers) were distributed for tree fiber production as vegetative propagation, which were applied to make Japanese paper (Miyazaki 1697; Okura 1946). Since then, vegetative propagation of elite landraces has been fully understood. In addition, chloroplast INDELs showed polymorphism within some elite lines, such as Nasu, Akaso, and Aoso (Table S3).

Kuo et al. (2022) reported Korean and Japanese cultivated paper mulberry such as Daknamu and Kozo. Six plastid genomes were aligned in the study. The results suggested that Daknamu has multiple maternal origins, and Kozo has a single maternal origin consistent with *B. monoica*. Hybridization was presumed to have occurred rarely, and it was unidirectional. Plastid haplotypes using *ndgF‐rpl32* resulted in a single haplotype in Kozo, which is shared with *B. monoica* in South Korea and *B. monoica* in Japan. Our marker system, cpINDELs, suggested that some elite lines belong to different maternal types (Table 1, Tables S1 and S3). In this study, complete chloroplast genome sequences of elite cultivars were determined. Potential polymorphic markers were suggested from sequence alignments for genetic discrimination. The polymorphisms indicated that their major variations resulted not only from a high mutational rate but also from the introduction of different maternal donors.

Few nuclear genetic markers have been developed to trace genetic lineages spread out around the Pacific Ocean because they involve nonmodel plant species such as paper mulberry (Peñailillo et al. 2016). The MIG‐seq marker system based on ISSR can be applied to nonmodel organisms. Although the power of the MIG‐seq marker system is in supplying high numbers of loci, these are always accompanied by an issue of missing data. In this paper, we tried to obtain a more complete dataset, and there are still isolated hypervariable maternal lineages among Japanese Kozo. This finding suggested that we can identify different lineages from archaeological DNA data if available. However, it also suggested that it may be hard to detect the true parental lines of donated fiber resources used to make historical washi paper. Such variable polymorphisms may be applicable for the evaluation of genetic diversity of paper fiber resources.

## 5 Conclusions

By analyzing chloroplast DNA sequence data, the cpINDELs markers developed in this study enabled us to distinguish 15 chloroplast genome types among Kozo landraces in Japan regardless of geographical origins. A phylogenetic tree drawn using whole chloroplast genomes demonstrated that Kaname is positioned far from Aoso, Akoso, Nasu, and Taori. The nuclear genome analysis indicates that there are two groups in which samples collected in Saga prefecture formed a distinct group. Most of the samples collected in Ibaraki were close to Nasu, whereas samples collected from Hyogo were close to Akaso and Aoso. Our findings provide the current state of the Kozo landraces in Japan, determining the genetic structure and origin of plant materials that have been used to make Japanese traditional papers.

## Author Contributions

**Dinh Thi Lam:** investigation (equal), methodology (equal), visualization (equal), writing – original draft (equal), writing – review and editing (equal). **Katsunori Tanaka:** software (equal). **Akihiko Takashima:** resources (equal). **Ayako Shibutani:** conceptualization (equal), funding acquisition (lead), resources (equal). **Ryuji Ishikawa:** conceptualization (equal), data curation (equal), formal analysis (equal), investigation (equal), methodology (equal), project administration (equal), resources (equal), software (equal), writing – original draft (lead), writing – review and editing (lead).

## Conflicts of Interest

The authors declare no conflicts of interest.

## Supporting information


**Figure S1.** Phylogenetic tree constructed using the neighbor‐joining method and measured nucleotide distances using the Jukes‐Cantor method (replicates were performed 1000 times for bootstrap analysis). The control was *B. papyrifera* .

**Table S1.** List of materials applied to MIG‐seq.

**Table S2.** cpINDEL to identify preliminary maternal origins.

**Table S3.** Polymorphism detected by newly developed cpINDEL markers.

## Data Availability

All data generated or analyzed during this study are included in this published article, its supplementary information files, and Raw MIG‐seq data are deposited at the DDBJ Sequence Read Archive (DRA) with accession numbers DRA rishikawa‐0001 (Submission), PSUB024808 (BioProject), SSUB031924(Biosample).
