# Supplementary figures and images for: Genetic Diversity on Farm in Japanese Paper Mulberry
Source: Ecol Evol. 2025 Jan 9;15(1):e70828. doi: 10.1002/ece3.70828 (PMC11717549; doi:10.1002/ece3.70828)

## Slide 1
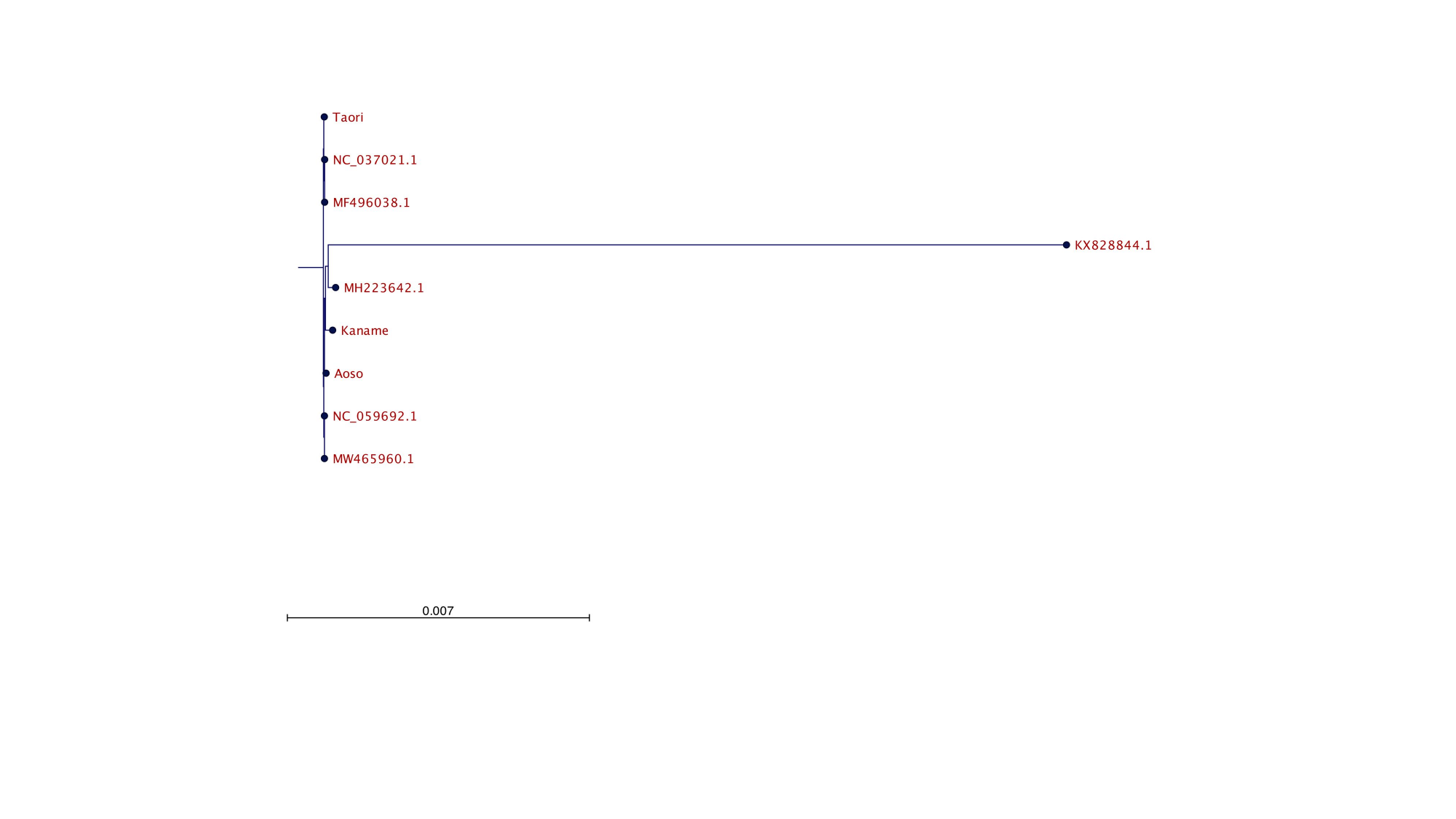

Supplement: Supplementary file 1 — Figure S1. Phylogenetic tree constructed using the neighbor‐joining method and measured nucleotide distances using the Jukes‐Cantor method (replicates were performed 1000 times for bootstrap analysis). The control was B. papyrifera . [file ECE3-15-e70828-s003.pptx]
